# Development and Validation of a questionnaire on human dignity in nursing cares: an exploratory sequential mixed study

**DOI:** 10.17533/udea.iee.v42n2e05

**Published:** 2024-07-04

**Authors:** Ali Dehghani

**Affiliations:** 1 Associate professor, Department of Community Health Nursing, School of Nursing, Jahrom University of Medical Sciences, Jahrom, Iran. Email: ali.dehghani2000@gmail.com. Corresponding Author. Jahrom University Iran

**Keywords:** personal respect, psychometrics, reproducibility of results, nursing care., respeto, psicometría, reproducibilidad de los resultados, atención de enfermería., respeito, psicometria, reprodutibilidade dos testes, cuidados de enfermagem

## Abstract

**Objective.:**

The current study aimed to develop and validate of human dignity questionnaire in nursing care.

**Methods.:**

The present research is a sequential exploratory mixed method study. The questionnaire was developed and validated in three phases: (1) the concept of human dignity was defined through conventional content analysis qualitative approach, (2) early items of questionnaire was generated according to findings of the first phase, (3) validation of the questionnaire was evaluated using face, content and construct validity as well as reliability. The study was conducted with the participation of 13 nurses in the qualitative section and 203 nurses in the quantitative section in teaching hospitals affiliated to Jahrom University of Medical Sciences (Iran).

**Results.:**

In the qualitative section, the definition and dimensions of the concept of human dignity in nursing care were discovered. In the quantitative section, the initial pool of items for the questionnaire of human dignity in nursing care was formed using the results of the qualitative section of the study and review of texts and related questionnaires. In factor analysis, four subscales including: respectful communication, equality of patient human value, preservation of privacy and patient-centered care were extracted by Eigen value above one. Internal consistency and stability of the questionnaire were calculated as 0.85 and 0.80, respectively, indicating an excellent reliability.

**Conclusions.:**

The 20-item developed questionnaire is valid and reliable for measurement of human dignity questionnaire in nursing cares.

## Resumen

**Objetivo.:**

Elaborar y validar un cuestionario sobre la dignidad humana en los cuidados de enfermería.

**Métodos.:**

Estudio exploratorio secuencial de método mixto. El cuestionario se desarrolló y validó en tres fases: (1) se definió el concepto de dignidad humana mediante un enfoque cualitativo de análisis de contenido convencional, (2) se generaron los primeros ítems del cuestionario de acuerdo con los resultados de la primera fase, (3) se evaluó la validación del cuestionario mediante la validez facial, de contenido y de constructo, así como la fiabilidad. El estudio se llevó a cabo con la participación de 13 enfermeras en la sección cualitativa y 203 enfermeras en la sección cuantitativa en hospitales docentes afiliados a la Universidad de Ciencias Médicas de Jahrom (Irán).

**Resultados.:**

En la fase cualitativa emergieron las dimensiones del concepto de dignidad humana en los cuidados de enfermería. En la sección cuantitativa, el grupo inicial de ítems para el cuestionario de dignidad humana en los cuidados de enfermería se formó utilizando los resultados de la sección cualitativa y de la revisión instrumentos relacionados. En el análisis factorial, se extrajeron cuatro subescalas con un valor Eigen superior a uno: comunicación respetuosa, igualdad del valor humano del paciente, preservación de la intimidad y cuidados centrados en el paciente, consistencia interna y la estabilidad del cuestionario se calcularon en 0.85 y 0.80, respectivamente, lo que indica una excelente fiabilidad.

**Conclusión.:**

El cuestionario de 20 ítems desarrollado es válido y fiable para medir la dignidad humana en los cuidados de enfermería.

## Resumo

**Objectivo.:**

Desenvolver e validar um questionário sobre a dignidade humana nos cuidados de enfermagem.

**Métodos.:**

Estudo exploratório sequencial misto de métodos exploratórios. O questionário foi desenvolvido e validado em três fases: (1) o conceito de dignidade humana foi definido utilizando uma abordagem de análise de conteúdo qualitativa convencional, (2) os primeiros itens do questionário foram gerados com base nos resultados da primeira fase, (3) a validação do questionário foi avaliada através da validade de face, de conteúdo e de construção, bem como da fiabilidade. O estudo foi realizado com a participação de 13 enfermeiros na secção qualitativa e 203 enfermeiros na secção quantitativa em hospitais universitários filiados na Universidade de Ciências Médicas de Jahrom (Irão).

**Resultados.:**

Na fase qualitativa, emergiram as dimensões do conceito de dignidade humana nos cuidados de enfermagem. Na secção quantitativa, o conjunto inicial de itens para o questionário sobre a dignidade humana nos cuidados de enfermagem foi formado utilizando os resultados da secção qualitativa e a revisão de instrumentos relacionados. Na análise fatorial, foram extraídas quatro subescalas com um valor Eigen superior a 1: comunicação respeitosa, igual valor humano do doente, preservação da privacidade e cuidados centrados no doente. A consistência interna e a estabilidade do questionário foram calculadas em 0.85 e 0.80, respetivamente, indicando uma excelente fiabilidade.

**Conclusão.:**

O questionário de 20 itens desenvolvido é válido e fiável para medir a dignidade humana nos cuidados de enfermagem.

## Introduction

Human dignity is the value that belongs to every human being simply by virtue of being human. Dignity is a central concept in nursing care^1^ and the maintenance of dignity has become an important aim in nursing care of patients.^2^ The concept of human dignity in nursing was first introduced in the ethical charter by the American Nurses Association as follows: “nurses must act with love and respect in all their professional relationships, taking into account their value and dignity”.^3^ In the Iranian nursing ethics, which was approved in 2012, the preservation of dignity and human dignity of patients has been emphasized as the first value concept.^4^ The fact that patients are one of the most vulnerable social groups doubles the importance of paying attention to maintaining their human dignity in hospital.^5^ In care settings, there are many situations that can potentially threaten patients' human dignity. In situations such as taking care of patients, maintaining privacy, physical examinations, introducing patients, lack of attention to people's appearance, nurse and patient not being the same sex, mixed section, neglecting to cover the patient's body, etc., the dignity of patients may be threatened. Therefore, it is necessary that the human dignity of patients in nursing care constantly considered by nurses.^6^ Research in this field also shows that the concept of human dignity is a global concern for nurses and most have emphasized the need for more research to identify the factors preservation of dignity and periodically assessment the human dignity of patients in the hospital.^7^^,^^8^

A review of studies shows that there are few questionnaires to assess the observance of human dignity in nursing care. Among these cases, the patient dignity inventory (PDI) is that developed by Chchinovo in 2008 to measure the sources of distress related to patient’s dignity nearing the end-of-life stage with 25 items and 5 dimensions of symptom distress, existential distress, dependency, peace of mind, and social support. This questionnaire examines the sources of distress related to the patient's dignity in palliative care (patients the end-of-life stage) and its items are not specific to the observance of human dignity in nursing care.^9^The above questionnaire, which was designed based on a review of texts, did not use the views and experiences of nurses to produce questionnaire items. It is also different from the conditions of nurses and the health care system in Iran in terms of context and therefore cannot accurately measure the level of observance of human dignity in nursing care, especially in Iran. Other questionnaires in this field are researcher-made scale in which the psychometric steps not done and only collecting items using reviewing the texts and assessing the content validity and internal consistency.^10^^,^^11^Therefore, since the concept of human dignity is a context-dependent concept^5^^,^^12^ and changes according to different moral, customary, religious, cultural and social conditions of the health care system in each country, there is a need for a specific questionnaire with socio-cultural relevance to be able to measure the intended concept accurately. Hence, the present study was conducted with the aim of development and psychometric assessment of a questionnaire to assess the observance of human dignity in nursing care using a combined qualitative and quantitative study.

## Methods

**Figure 1:**
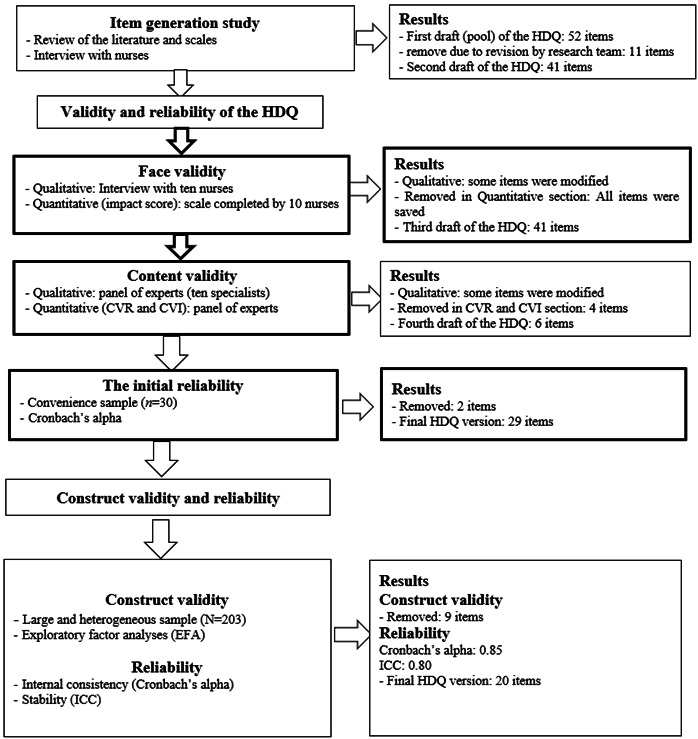
Flow diagram of the development and validation of the human dignity questionnaire (HDQ)

The present research is a sequential combined exploratory study that was conducted in two parts of qualitative and quantitative (Figure 1). Data were collected from January to October 2021 in teaching hospitals affiliated to Jahrom University of Medical Sciences. The present combined study was performed with the following phases:^13^ 1-Define and explain the concept of human dignity using a qualitative study; 2- Design the initial items of the questionnaire using the results of the first step and review the related questionnaires; and 3- Checking the validity and reliability of the questionnaire items.

### The first phase

In this phase, the concept of human dignity was defined and its dimensions were explored using qualitative content analysis. In this phase, the conventional content analysis method was used. In this approach, researchers interpret results by presenting data in words and themes, which involves understanding, interpreting, and conceptualizing the underlying meaning of qualitative data. In the conventional content analysis approach, the codes and categories of the concept are obtained directly from the interview.^14^ Participants in the qualitative section included 13 nurses whose views on the concept of human dignity were collected through semi-structured face-to-face and in-depth interviews. The duration of each interview was between 40 - 60 minutes. Sampling from nurses in this section continued until data saturation so that no new data and category of interviews were obtained. Inclusion criteria for nurses included the ability to speak in Persian language, at least 2 years of clinical experience in hospital wards and willingness to participate in the study. The research environment was real field, so the interviews were conducted in the nurses' rest room at the hospital with the prior consent of the participants. In the present study, a series of pre-designed questions related to the research topic were used to guide the interview process. The interview started with more general questions and with the progress of the interviews and the simultaneous analysis of the data as well as the type of answers of the participants, more detailed questions were asked. Sample interview questions were as follows. 1) Can you tell me what ethical points you pay attention to in caring for patients? 2) What do you mean by human dignity? What does it matter? 3) What experiences (good and bad experiences) do you have in the field of respecting the human dignity of patients in nursing care? 4) How can nurses respect the human dignity of patients in nursing care? To analyze the findings in this section, the approach of qualitative content analysis by Granheim and Landman method was used.^15^ First, the interviews were implemented in MAXQDA version 10 software for data management. Then interviews text read line by line and paragraph by paragraph several times to gain a general understanding of the concept. The initial codes were extracted. In the next step, sub-categories and categories were obtained based on the differences and similarities of the extracted codes. At the end of this phase, the definition and dimensions of the concept of human dignity in nursing care were discovered.

### The second phase

In this phase, the initial pool of items for the questionnaire of human dignity in nursing care was formed using the results of the qualitative section of the study and review of texts and related questionnaires.

### The third phase

In this phase, the psychometric steps of the scale were performed on the initial items of the questionnaire. Psychometric properties included face validity, content validity, structural validity and reliability.

***Face validity*.** The face validity of the questionnaire was performed using two methods of qualitative and quantitative. In the qualitative section, face-to-face interviews were conducted with 10 nurses regarding the difficulty, appropriateness and ambiguity of the items. Then their opinions were applied on the items of the questionnaire. In the quantitative part, in order to obtain the importance of the items, the impact factor of the items was used.^16^^,^^17^ For this purpose, 10 participants will score the importance of the items based on the 5-point Likert scale as completely important (5), somewhat important (4), moderately important (3), slightly important (2), and not important at all (1).^16^^,^^17^The researcher then will calculate the impact score of each item separately using the following formula: Impact Score = Frequency (%) × Importance; Frequency (%) refers to the number of persons who scored 4 and 5 on each item, and importance refers to the mean score of importance based on the Likert spectrum. Items were retained with an impact factor score greater than 1.5 for analysis of other psychometric steps.^16^

*Content validity.* The content validity of the questionnaire was performed using both qualitative and quantitative methods. In the qualitative section, the researcher asked a panel consisting of experts to give their opinions in terms of the grammar, proper word placement, proper item placement, and appropriate scoring after studying the scale. The scale was modified according to them. In the quantitative section, the content validity ratio (CVR) and content validity index (CVI) was determined. The CVR evaluate the necessity for items of scale from the perspective of the expert’s panel. For this aim, the experts were asked to assess each item based on the 3-point scale (necessary, useful but unnecessary, and unnecessary). Then CVR was calculated based on the following formula: CVR = (Ne - N/2) ÷ N/2; where N is the total number of experts and Ne is the number of experts who choose the necessary option.^16^^,^^17^ For determine of CVR from 10 experts was used. Then the decision was made based on Lawshe^18^ and the modified table by Ayre and John Scally.^19^CVI checked out in the 4-point Likert scale for each item by 10 experts (e.g. 1: irrelevant; 2: somewhat relevant; 3: relevant; and 4: quite relevant). Then, the CVI score calculated through combining the agreement scores for each item ranked 3rd and 4th by the total number of experts. Values ranged from 0 to 1, and when CVI > 0.79, the item was relevant, from 0.70 to 0.79, the item needed revisions, and if the item value was below 0.70, was eliminated.^20^

***The initial reliability*.** The initial reliability of the questionnaire was performed before the construct validity to determine the correlation between the items and the whole questionnaire. Calculating the initial reliability by deleting or correcting inappropriate items facilitates factor analysis and improves the reliability of the questionnaire. In this section, reliability was performed by calculating Cronbach's coefficient alpha and completing a questionnaire by 30 nurses.^16^^,^^17^

***Construct validity*.** The construct validity of the questionnaire was assessed using exploratory factor analysis.^16^^,^^21^

***The final reliability*.** In this section, two methods of internal consistency and stability were used. To determine the internal consistency, the questionnaire was completed by 30 nurses and then Cronbach's coefficient alpha was calculated. Cronbach's coefficient alpha above 0.7 indicated acceptable internal consistency. ^16^^,^^17^ Test-retest method was used to determine the stability. For this purpose, the questionnaire was completed twice at intervals of two weeks by at least 30 nurses. Then the intra-class correlation coefficient (ICC) was calculated. Intra-class correlation coefficient above 0.7 indicated acceptable stability.^16^^,^^22^

### Ethics approval and consent to participate

The research was approved by the Ethics Committee at Jahrom University of Medical Sciences (ethical number: IR.JUMS.REC.1399.019). The research was conducted in accordance with the ethical principles of the Declaration of Helsinki and the guidelines of the Iranian Ministry of Health and Medical Education. All participants signed a written informed consent form.

## Results

The results of the study are presented in three phases (Figure 1):

### The first phase

Based on the experience of nurses using conventional content analysis and literature review, the dimensions of the concept of human dignity in nursing care included respectful communication, equality of patient human value, preservation of privacy and patient-centered care. 

### The second phase

Based on the categories extracted in the qualitative stage as well as reviewing the related questionnaires in this field, the initial pool of items was designed to development the questionnaire. Thus, 52 items were formed in the primary pool of items. After review by the research team, 11 items were removed due to overlap.

### The third phase

***Face validity*.** In this section, all items had an impact factor of 1.5. Therefore, no item was deleted in this section and a number of items were reviewed.

*Content validity.* No item was removed in the qualitative review of content validity. In the quantitative part, 4 items were removed due to CVR value less than 0.75 and 6 items were removed due to CVI value less than 0.79*.*

*The initial reliability.* The internal consistency of the whole questionnaire with Cronbach's coefficient alpha in a sample of 20 nurses was calculated 0.79. The number 2 item was removed using inter - item correlation due to a correlation of less than 0.3 with the whole questionnaire.

### Construct validity

In this section, the developed questionnaire was completed by 203 nurses with the remaining 29 questions. The demographic characteristics of the nurses are given in Table 1. Then, to determine the number of factors, exploratory factor analysis was performed. KMO statistic was performed for sampling adequacy which was 0.9231. The Bartlett sphericity test was significant (*p* = 0.001). To extract the factor structure of the questionnaire, principal component analysis and varimax rotation were performed with Eigenvalue> 1. The factor loading of each item was considered at least 0.5. ^16^ Of the initial 29 items, 9 items were omitted due to a factor loading of less than 0.5. Thus, the final questionnaire with 20 items and 4 factors including respectful communication (5 items), equality of patient human value (4 items), preservation of privacy (4 items) and patient-centered care (7 items) was obtained (table 2). The variance of 4 factors was 56.76%. **Scoring.** The items of the HDQ were rated on 5-point Likert-type scale from 1 to 5, 1 = never, 2 = rarely, 3 = sometimes, 4 = often, and 5 = always. The range of scores of this questionnaire is 20-100, so that a higher score indicates more observance of human dignity in nursing care.

***The final reliability*.** The internal consistency of the HDQ for 20 items using Cronbach's coefficient alpha was obtained 0.85. The stability of the HDQ using the ICC was calculated 0.80, which indicates that the HDQ has good stability over time. Internal consistency and ICC for 4 factors are listed separately in Table 3.

**Table 1:** Demographic characteristics of the nurses in the quantitative section of the study

Variable	Sub-variables	***n* (%)**
Gender	Male	73 (35.97)
	Female	130 (64.03)
Marriage	Single	39 (19.2)
	Married	152 (74.88)
	divorced	12 (5.92)
Education	Bachelor of Nursing	175 (86.2)
	Master of Nursing	28 (13.8)
Age	Mean ± Standard deviation	34.05 ± 5.67
Work experience	Mean ± Standard deviation	9.86 ± 3.10

**Table 2:** Factors, items and factor loadings of the human dignity scale (HDQ-20)

Subscales	Items	Factors		
		1	2	3	4
Respectful communication	I treat the patient with respect.	0.601	0.321	0.343	0.232
	I consider having a good ethic in caring for the patient's rights and I adhere to it.	0.589	0.012	0.399	0.211
	I introduce patients to colleagues by name (not bed number).	0.599	0.287	0.323	0.123
	I make eye contact when talking to patients.	0.564	-0.014	0.321	0.234
	Before any care, I introduce myself to the patient.	0.532	0.146	0.156	0.121
Equality of patient human value	I see each patient as a unique person with equal human value.	0.278	0.754	0.265	0.278
	I accept the patient as a member of my family.	0.324	0.721	0.213	0.232
	I respect for rationality and patients' choices.	0.345	0.678	0.165	0.043
	In caring for the patient, nationality, race, culture, etc. are of equal importance to me.	0.235	0.611	-0.012	0.235
Preservation of privacy	In order to preservation the privacy of patients, I pay attention to their coverage during examinations, injections, etc.	0.290	0.343	0.632	0.190
	I do not tell patients' private secrets in front of others.	0.367	0.345	0.598	0.343
	I try to take care of female patients by a female nurse and male patients by a male nurse.	0.239	0.123	0.578	0.343
	Before entering the patient room, I knock on the door and enter the room with coordination.	0.354	0.278	0.511	0.231
Patient-centered care	I pay attention to patients' priorities regarding how and quality of care.	0.254	0.354	0.123	0.755
	I respect the patient's choice regarding the caregiver.	0.324	0.142	0.342	0.721
	I involve patients in self- care.	-0.243	0.234	0.361	0.655
	I respect patients' independence in treatment decisions.	0.288	0.288	0.195	0.611
	Based on the beliefs and religious principles of the patients, I provide them with the necessary facilities.	0.239	0.387	0.188	0.634
	I consider myself responsible for responding to the concerns and concerns of patients and their companions.	0.154	0.321	0.018	0.443
	I provide sufficient and required information to patients before performing care.	0.129	0.324	0.234	0.524
Eigenvalue		6.321	4.876	3.256	2.189
Percentage of variance		18.176	15.453	12.214	10.917

**Table 3:** The Cronbach's alpha and The ICC values of the HDQ scale

Factors	Subscales	Number of items	Internal consistency	Stability
1	Respectful communication	5	α = 0.88	ICC = 0.84
2	Equality of patient human value	4	α = 0.83	ICC = 0.77
3	Preservation of privacy	4	α = 0.78	ICC = 0.79
4	Patient-centered care	7	α = 0.89	ICC = 0.86
Total	HDQ	20	α = 0.85	ICC = 0.80

## Discussion

The present study attempted to develop and validate a scale for human dignity in nursing cares. The findings show that the HDQ scale is a reliable and valid scale for the evaluation of the human dignity. The HDQ scale showed that the concept of human dignity in nursing care has many dimensions including respectful communication, equality of patient human value, preservation of privacy and patient-centered care. One of the dimensions of the HDQ scale was the respectful communication with 5 items. The Attributed Dignity Scale by Jacelon *et a*l.,^23^had 23 items and three dimensions of self-value, behavioral respect-self, and behavioral respect-others. In this questionnaire, in order to observance of human dignity, the dimensions of respect for the patient have been considered, which is consistent with the dimension of respectful communication in the HDQ scale. In the study by Henderson et al., The issue of respectful communication was noted that most nurses do not spend enough time communicating with the patient and are busy with other tasks or looking at equipment and do not make proper eye contact with the patient, so that this neglect and indifference is effective in creating a sense of worthlessness and reduce human dignity in patients.^24^

The second dimension of the HDQ scale was the equality of patient human value with 4 items. The data of this dimension show that all human regardless of race, ethnicity, culture, nationality, skin color, etc., are equal and there should be the equal viewpoint of all patients in care. According to the nurse’s viewpoint of participating in the study of Walsh and Kowanko^25^ the object-oriented view of the patient is a threat to her human dignity, and all patients deserve respectful communication and the preservation of human dignity because of their equal intrinsic value. The results of this study also showed that from the nurses' point of view, issues related to the patient's dignity include the patient's body cover, patient's privacy, attention to emotions, time allocation, considering the patient as a human being, showing respect to the patient. Issues related to the patient's dignity from the patients' point of view include not exposing the patient's body, allocating time for the patient, being seen as a human being, thanking and appreciating, and paying special attention to the patient.^25^According to the results of the study of Manoukian *et al*.,^26^The personality traits of the medical-care staff, their individual values and beliefs about the value and human status of the patient are among the factors affecting the maintenance of human dignity of patients. The results of Cheraghi *et a*l.^27^ qualitative study based on patients' perspectives showed that the patient's dignity is integrated into two categories “exigency of respecting human nobility’’ and ‘‘providing person-centered care”, which is consistent with the dimensions of " equality of patient human value " and "patient-centered care" extracted in the present study.

The third dimension of the HDQ scale was the preservation of privacy with 4 items. Previous studies demonstrated that nurses acknowledged that protecting patients’ privacy is a necessary factor of preserving their human dignity, and that dignity can be lost when physical or other personal boundaries are transgressed. The results of a qualitative study by Papastavrou *et al*.^28^ showed that one of the dimensions of the patient dignity from the perspective of nursing students is privacy and confidentiality. Also, the results of a qualitative study by Bagheri *et al*.^29^ showed that communication and observance of privacy are effective factors on the dignity of patients with heart failure. In the qualitative study of Manoukian *et al*.,^5^one of the important dimensions of maintaining patient dignity, which is emphasized in the statements of participants, is confidentiality and keeping patient secrets, because the issue of patient privacy and information privacy as one of the accepted principles among patients and health care providers. 

The fourth dimension of the HDQ scale was the patient-centered care with 7-item. Patients’ dignity has been intertwined with the concept of caring and acknowledged by our caring capacity. Caring is an ethical attitude and is a characteristic of humans by acknowledging another person’s self-determined ends as one’s own to maintain or promote them.^27^^,^^30^ According to Carrillo et al., patient-centered care is derived from study in cross-cultural care.^31^ Wainwright and Gallagher stressed that it is essential for nurses to pay more attention to what it means for patients to be respected and provided proper culturally sensitive care.^32^ This refers to ‘‘providing patient-centered care,’’ where different features of a person’s life contributing to diverse backgrounds are acknowledged.^33^ Similarly, in the items of patient-centered care dimension in the HDQ scale, the issue of patients' beliefs and religion is mentioned, which should be considered by nurses as one of the dimensions of care. Developing a questionnaire for human dignity in nursing cares can pave the way for further plans and measures. This study is also based on a combination of methods; therefore, it can support the integration of different and even contradictory approaches and methods. Collecting qualitative and quantitative data will help better perceive nurses experiences regarding observance of human dignity in the patients. 

Limitations of the study. This study has also limitation, including sampling in only one city of Iran. To mitigate this limitation, sampling was done with maximum variation. Another limitation of this study is the lack of interviewing and soliciting opinions from patients. 

Conclusion. This study led to the creation of a valid and reliable questionnaire for assessing patients' human dignity in nursing care. The final version of the HDQ has 20 items in four domains including: respectful communication, equality of patient human value, preservation of privacy, and patient-centered care. This questionnaire is a consistent, simple, valid, reliable and context-based scale, which can be used in a variety situations of the clinical care. The Iranian healthcare and hygiene managers can periodically measure the patients' human dignity in nursing care and design and implement a care plan based on HDQ scale that includes the ethical principles related to human dignity.
